# *Toxoplasma gondii* Infection in the United States, 1999–2000

**DOI:** 10.3201/eid0911.030098

**Published:** 2003-11

**Authors:** Jeffrey L. Jones, Deanna Kruszon-Moran, Marianna Wilson

**Affiliations:** * Centers for Disease Control and Prevention, Atlanta, Georgia, USA; † National Center for Health Statistics, Centers for Disease Control and Prevention, Hyattsville, Maryland, USA

**Keywords:** *Toxoplasma gondii*, toxoplasmosis, prevalence, United States

## Abstract

Infection with *Toxoplasma gondii* can lead to congenital and acquired disease, resulting in loss of vision and neurologic illness. We tested sera collected in the National Health and Examination Survey (NHANES) from 1999–2000 for *T. gondii*–specific immunoglobulin G antibodies and compared these results with results from sera obtained in the NHANES III survey (1988–1994). NHANES collects data on a nationally representative sample of the U.S. civilian population. Of 4,234 persons 12–49 years of age in NHANES 1999–2000, 15.8% (age-adjusted, 95% confidence limits [CL] 13.5, 18.1) were antibody positive; among women (n=2,221) 14.9% (age-adjusted, 95% CL 12.5, 17.4) were antibody positive. *T. gondii* antibody prevalence was higher among non-Hispanic black persons than among non-Hispanic white persons (age-adjusted prevalence 19.2% vs. 12.1%, p=0.003) and increased with age. No statistically significant differences were found between *T. gondii* antibody prevalence in NHANES 1999–2000, and NHANES III. *T. gondii* antibody prevalence has remained stable over the past 10 years in the United States.

*Toxoplasma gondii* is a ubiquitous protozoan parasite of warm-blooded animals. However, only members of the cat family (*Felidae*) are definitive hosts for the organism, which is shed in their feces for several weeks after the organism has completed a sexual cycle in their intestinal epithelial cells. Infection in humans generally occurs either by ingesting viable tissue cysts in raw or undercooked meat or by ingesting oocysts shed in the feces of a cat. After acute infection, *T. gondii* continues to exist in tissue cysts in humans, particularly in the muscles and brain. Tissue cysts are long-lived and not associated with disease. However, in people with immunodeficiencies such as AIDS or malignancies, rupture of cysts results in disease reactivation, including encephalitis or disseminated toxoplasmosis. Immunoglobulin (Ig) G antibodies to *T. gondii* appear early, reach a peak within 6 months after infection, and are detectable for life.

When a pregnant woman is infected for the first time, and the infection spreads to the fetus, congenital *T. gondii* infection may be clinically apparent in the neonate in the first months of life or later during infancy, childhood, or adolescence (i.e., cause neurologic or eye damage) or may remain subclinical. An estimated 400 to 4,000 cases of congenital toxoplasmosis occur each year in the United States (*1*). In an analysis of data from a large HIV-infected cohort, toxoplasmosis was found to be the most frequent severe neurologic infection among persons with AIDS in the United States, even after the advent of highly active antiretroviral therapy (*2*).

The United States Department of Agriculture (USDA) estimates that one half of *T. gondii* infections in the United States are caused by ingestion of raw or undercooked infected meat (*3*). A community-based study in Maryland, comparing persons who did not eat meat with those who did eat meat, supports the USDA estimate (*4*). In 1999, Mead and colleagues estimated that of the 750 deaths caused by toxoplasmosis each year, one half were the result of eating raw or undercooked meat, making toxoplasmosis the third leading cause of foodborne deaths (*5*).

To present the prevalence of infection in the U.S. population, we report the *Toxoplasma*-specific IgG results of the National Health and Nutrition Survey (NHANES) conducted in 1999–2000 and compare the prevalence of *Toxoplasma* IgG antibody seropositivity during these years to the prevalence observed previously in NHANES III 1988–1994. In the NHANES III national probability sample, 22.5% of 17,658 persons >12 years of age had *Toxoplasma*-specific IgG antibodies, indicating that they had been infected with the organism (published prevalence was age-adjusted to the 1980 U.S. population; prevalence for same population age-adjusted to the 2000 U.S. population is 23.6%) (*6*).

## Methods

### NHANES Samples

Beginning in 1999, NHANES became a continual survey. Each survey year is based on a nationally representative sample of the U.S. civilian noninstitutionalized population, selected with a stratified, multistage, probability cluster design. Data are collected on health measures and conditions through household interviews, standardized physical examinations, and blood samples obtained at mobile examination centers. The procedures followed to select the sample and conduct the interviews and examinations are similar to those of previous NHANES surveys (*6*). The continual NHANES is released in 2-year groupings (cycles). Two or more years of data are necessary to have adequate sample sizes for subgroup analyses. This report is based on the first 2 years of the continual NHANES (1999–2000).

Serum samples were available for testing for *T. gondii* antibodies from a nationally representative sample of persons 6–49 years of age in NHANES 1999–2000 and from persons >12 years of age in NHANES III. To compare NHANES 1999–2000 with NHANES III, the principal analyses are limited to the overlapping age groups (i.e., 12–49 years of age) and stratified on variables previously examined in NHANES III (*6*). Age was grouped as 12–19, 20–29, 30–39, and 40–49 years. In NHANES III, serum specimens were also available from a limited number of children 6–11 years of age (n=1,819, 48% of children sampled in this age range). Although the NHANES III data in this 6–11 year age group cannot be considered nationally representative because of the low response rate, we present the previously calculated prevalence (*6*) for this group in our results because these are the only U.S. data available for this age.

Race/ethnicity was defined as self-reported non-Hispanic white, non-Hispanic black, or Mexican American (in NHANES III, oversampling of Mexican Americans was conducted to ensure adequate sample size for this group). In NHANES 1999–2000, the race/ethnicity variable used was the one most consistent with the NHANES III race/ethnicity coding. The NHANES 199–2000 sample size was not sufficient to stratify by other racial and ethnic groups; however, these groups were included in the estimates given for the total study population.

Informed consent was obtained from patients or their parents or guardians, and human subjects review guidelines of the U.S. Department of Health and Human Services were followed in the conduct of this research.

### Laboratory Testing

NHANES 1999–2000 specimens were tested by using the Platelia Toxo-G enzyme immunoassay kit (BioRad, Hercules, CA), according to the manufacturer’s instructions. Results were reported in international units (IU); samples with >10 IU were considered positive for *T. gondii* IgG antibodies. NHANES III serum specimens had been tested with the same kit, however >6 IU was used as a cutoff for seropositivity. As a result of minor changes in the kit, the company changed the IU cutoff value for kits used to test the 1999–2000 sera. However, test positivity should be considered to be equivalent for both studies, regardless of the cutoff values.

### Statistical Analysis

Prevalence estimates were weighted to represent the U.S. population, to account for oversampling in specific demographic subgroups, and to account for nonresponses to the household survey and to the physical examination. Estimates and standard errors were calculated by using SUDAAN (*7*). Standard errors for NHANES 1999–2000 were estimated by means of the delete 1 jackknife (JK1) method (*8*). In previous NHANES surveys, the Taylor series linearization method was used to estimate standard errors. These standard errors account for the sample weights and complex sample design. Prevalence estimates were age-adjusted by the direct method to the 2000 U.S. population for both NHANES III and NHANES 1999–2000 when seroprevalence was compared across population subgroups. Ninety-five percent confidence intervals were calculated by using a t-statistic; p values testing the significance of the difference in prevalence between NHANES III and NHANES 1999–2000 were obtained by using a t-statistic with the combined standard error.

## Results

Of the 4,875 persons 12–49 years of age who were selected for NHANES 1999–2000, a total of 4,602 (94.4%) persons were interviewed and underwent physician examination; of these, 4,234 persons (86.9% of those selected) had serum specimens tested for *T. gondii* antibodies. In NHANES 1999–2000, the percentage of those tested for *T. gondii* IgG antibodies among those examined did not vary by race/ethnicity, sex, or country of birth. Some variability existed, but no consistent trend, with age in the percentage of persons with sera tested among those examined (range 91% to 94%). Of the 4,234 persons tested for *T. gondii* IgG antibodies, 638 (15.8%, age-adjusted, 95% confidence limits [CL] 13.5, 18.1) were antibody positive. Among women (n=2,221), 14.9% (age-adjusted, 95% CL, 12.5, 17.4) were antibody positive. *T. gondii* antibody prevalence for men was similar to that for women (age-adjusted, 16.7% vs. 14.9%, respectively, p=0.28), higher among non-Hispanic blacks than among non-Hispanic whites (age-adjusted, 19.2% vs. 12.1%, p=0.003), and higher as age increased (Table). The *T. gondii* antibody prevalence was also higher in Mexican Americans than in non-Hispanic whites, but the difference was not statistically significant (16.8% vs. 12.1%, p=0.051). In NHANES III, the overall age-adjusted seroprevalence was similar for men and women and higher in Mexican Americans than in non-Hispanic whites in the age range examined for this article.

**Table:** Comparison of *Toxoplasma gondii* immunoglobulin G antibody seroprevalence in NHANES 1999–2000 and NHANES III (1988–1994)^a,b,c^

	NHANES 1999–2000			NHANES III		
	N^d^	Prevalence	95% CL	N^d^	Prevalence	95% CL
Total	4,234	15.8	13.5, 18.1	11,132	16.0	14.5, 17.5
Sex						
Male	2,013	16.7	13.6, 19.9	5,144	16.7	14.8, 18.6
Female	2,221	14.9	12.5, 17.4	5,988	15.3	13.5, 17.0
Race/ethnicity						
Non-Hispanic white	1,293	12.1	9.9, 14.4	3,304	14.3	12.5, 16.2
Non-Hispanic black	1,027	19.2	14.8, 23.6	3,674	18.0	16.1, 19.8
Mexican American	1,553	16.8	12.4, 21.1	3,661	18.3	16.7, 20.0
Age group						
12–19	2,105	9.3	6.4, 12.1	2,749	8.5	6.4, 10.5
20–29	735	13.4	10.1, 16.7	3,100	15.2	12.1, 18.3
30–39	726	18.1	14.7, 21.5	2,960	16.1	14.6, 17.6
40–49	668	20.4	15.7, 25.0	2,323	22.2	19.4, 25.0
Country of birth						
United States	3,211	12.2	10.0, 14.3	8,606	14.1	12.7, 15.5
Non-U.S.	995	32.8	27.3, 38.3	2,493	27.9	24.1, 31.7

^a^NHANES, National Health and Examination Survey. ^b^Sex, race/ethnicity, and total values are age-adjusted to the 2000 census estimated population, using the four age categories shown in the table. ^c^No statistically significant differences (p>0.05, t-statistic) existed between NHANES 1999–2000 and NHANES III across any subgroup in the table. ^d^Totals for the race/ethnicity or country of birth categories do not add up to the total number because of an “other” category for race/ethnicity (not shown) or because persons did not provide a response to country of birth questions.

No significant differences were found between NHANES 1999–2000 and NHANES III *T. gondii* antibody prevalences overall or in any of the sex, race, or age categories (Table, comparing values horizontally by rows). In NHANES 1999–2000, children 6–11 years of age had a *T. gondii* antibody prevalence of 8.0% (95% CL 4.5, 11.5, N=855) (data not shown in table). In NHANES III, the antibody prevalence for children 6–11 years of age was 5.2% (*6*), however, as noted in Methods, this estimate may be subject to nonresponse error (data not shown in table).

The *T. gondii* antibody prevalence was higher in persons born outside the United States than in U.S.-born persons for both NHANES 1999–2000 and NHANES III (age-adjusted, 32.8% vs. 12.2% and 27.9% vs. 14.1%, respectively, Table), but among persons born outside the United States seroprevalence did not differ significantly between NHANES 1999–2000 and NHANES III (p>0.05). In addition, the percentage of persons that were born outside the United States was not significantly different in NHANES 1999–2000 (16.3%, 95% CL 11.8%, 20.7%) compared with the percentage of persons born outside the United States in NHANES III (13.3%, 10.9%, 15.7%) (p>0.05).

## Discussion

We found an overall *T. gondii* IgG antibody prevalence of 15.8% among persons 12–49 years of age in 1999–2000, indicating that approximately 1 in 6 persons in this age group was infected with *T. gondii*. No significant changes in *T. gondii* seroprevalence occurred between 1988–1994 and 1999–2000 for the U.S. population as a whole or for any of the subgroups we examined. We had speculated that changes in meat production with lower levels of *T. gondii* in meat (*9*) might result in a reduction in the prevalence of *T. gondii* infection in the population. Perhaps the time was not sufficient for changes in meat production and consumption habits to have had an impact, or perhaps the expected declines in *T. gondii* infection occurred before NHANES III. The prevalence of *T. gondii* infection declined in U.S. military recruits, when 1965 data were compared with 1989 data (*10*) and in countries in Europe, such as France and Belgium, during similar periods (*11*).

Predicting future trends in *T. gondii* prevalence in the United States is difficult because we do not have a national estimate of what proportion of *T. gondii* infections are attributable to undercooked meat exposure or to cat feces, soil, or water exposure. A large European case-control study that examined these factors showed that undercooked meat accounted for the largest portion of risk for *T. gondii* infection (30%–60%, depending on location) (*12*). However, until researchers examine the risk factors for *T. gondii* infection in a case-control study throughout the United States, the most important U.S. risk factors and how to best focus preventive education messages cannot be determined.

In NHANES 1999–2000, the *T. gondii* antibody prevalence was higher among non-Hispanic black persons than non-Hispanic white persons. This finding may reflect immigration patterns from countries with higher rates of *T. gondii* infection or soil exposure and culinary practices among these different populations. The seroprevalence among persons born outside the United States tended to be higher in NHANES 1999–2000 than in NHANES III, and the percentage of persons born outside the United States tended to be higher in NHANES 1999–2000 than NHANES III, but these findings were not statistically significant. Clearly, in both NHANES III and NHANES 1999–2000 the seroprevalence is higher among persons not born in the United States than in U.S.-born persons. The NHANES 1999–2000 sample population is not large enough to stratify racial/ethnic groups by foreign-birth status and obtain accurate estimates; however, in a multivariate analysis reported previously that used NHANES III data (*6*), being born outside the United States was a significant risk factor for *T. gondii* seropositivity. However, race/ethnicity did not increase risk (using white non-Hispanic persons as the reference group).

NHANES gives representative estimates of prevalence for the U.S. population but is not designed to evaluate local *T. gondii* prevalence levels. Studies have indicated that *T. gondii* prevalence varies greatly in the United States (*10**,**13**,**14*); this local variation is most likely related to culinary practices, the ability of oocysts to survive in different climates, and the levels of immigration from areas of the world in which *T. gondii* infection is highly endemic. Nevertheless, NHANES produces useful surveillance data for tracking *T. gondii* prevalence over time in the United States. We will continue to monitor trends in this nationally representative survey.
